# Role of the CCL2-CCR2 axis in cardiovascular disease: Pathogenesis and clinical implications

**DOI:** 10.3389/fimmu.2022.975367

**Published:** 2022-08-30

**Authors:** Haixia Zhang, Ke Yang, Feng Chen, Qianqian Liu, Jingyu Ni, Weilong Cao, Yunqing Hua, Feng He, Zhihao Liu, Lan Li, Guanwei Fan

**Affiliations:** ^1^ First Teaching Hospital of Tianjin University of Traditional Chinese Medicine, National Clinical Research Center for Chinese Medicine Acupuncture and Moxibustion, Tianjin, China; ^2^ Hebei Key Laboratory of Integrated Traditional Chinese and Western Medicine for Diabetes and Its Complications, College of Traditional Chinese Medicine, North China University of Science and Technology, Tangshan, China; ^3^ Hubei Key Laboratory of Economic Forest Germplasm Improvement and Resources Comprehensive Utilization, Huanggang Normal University, Huanggang, China; ^4^ State Key Laboratory of Component-Based Chinese Medicine, Tianjin University of Traditional Chinese Medicine, Tianjin, China; ^5^ Key Laboratory of Pharmacology of Traditional Chinese Medical Formulae, Ministry of Education, Tianjin University of Traditional Chinese Medicine, Tianjin, China

**Keywords:** chemokine, CCL2, CCR2, inflammation, cardiovascular disease

## Abstract

The CCL2-CCR2 axis is one of the major chemokine signaling pathways that has received special attention because of its function in the development and progression of cardiovascular disease. Numerous investigations have been performed over the past decades to explore the function of the CCL2-CCR2 signaling axis in cardiovascular disease. Laboratory data on the CCL2-CCR2 axis for cardiovascular disease have shown satisfactory outcomes, yet its clinical translation remains challenging. In this article, we describe the mechanisms of action of the CCL2-CCR2 axis in the development and evolution of cardiovascular diseases including heart failure, atherosclerosis and coronary atherosclerotic heart disease, hypertension and myocardial disease. Laboratory and clinical data on the use of the CCL2-CCR2 pathway as a targeted therapy for cardiovascular diseases are summarized. The potential of the CCL2-CCR2 axis in the treatment of cardiovascular diseases is explored.

## Introduction

Cardiovascular disease is the world’ s leading cause of morbidity and mortality (1, 2). A rising body of evidence suggests that inflammation is a key contributor to cardiovascular disease (3, 4). Randomized clinical trials based on canakinumab (5) and colchicine (6) have evidenced the effectiveness of particular anti-inflammatory treatments in the field of cardiovascular disease prevention. In the course of inflammatory immune response, leukocytes are recruited to the site of injury. Chemokines and its receptors have been described as essential mediators that regulate leukocyte infiltration and migration to specific sites of inflammatory response (7). There is powerful proof that chemokine CCL2 and its receptor CCR2 function in cardiovascular diseases such as heart failure (8), atherosclerosis and coronary atherosclerotic heart disease (9), hypertension (10) and cardiomyopathy (11) (**Table 1**). In addition, high levels of circulating CCL2 are related to increased long-term cardiovascular mortality in people without significant cardiovascular disease (43).

**Table 1:** Summary of selected studies investigating the role of CCL2 & CCR2 in cardiovascular diseases.

Conditions	Experimental Model/Study population	Major findings related to CCL2/CCR2 axis		References
Heart Failure				
CHF	Rats with ACF	CCL2 contributed to the progression of cardiac decompensation and the development of CHF	(8)	
Hypertensive heart disease	Rats with suprarenal aortic constriction	CCL2-mediated macrophage aggregation acted on myocardial fibrosis *via* a TGF-β-mediated process	(12)	
Genetic HF	Des^-/-^ mice	Downregulation of CCR2, Arg1 and pro-fibrotic gene expression ameliorated poor cardiac remodeling, inflammation and failure	(13)	
End-stage HF	Hearts from patients with end-stage HF and in organ donors	First demonstrated the expression and protein localization of CCL2/CCR2 in human myocardium	(14)	
CHF	CHF patients	Serum CCL2 levels were positively related to the seriousness of symptoms as well as the degree of left heart insufficiency in patients with CHF	(15)	
Advanced HF	Advanced HF patients	CCL2 was significantly associated with poor prognosis in patients with advanced heart failure	(16)	
Atherosclerosis and coronary artery atherosclerotic heart disease				
Atherosclerosis	Rabbits with endothelial desiccation and atherogenic diet	CCL2-induced migration of monocytes to the vessel wall was a key activity contributing to the development of atherosclerosis	(17)	
Atherosclerosis	Diseased human arteries	CCL2 had a potential function in mediating mononuclear cell infiltration in the arterial wall	(18)	
Atherosclerosis	LDL-R^-/^-/CCL2^-/-^ mice with high cholesterol diet	CCL2 played a specific and essential role in the activation of atherosclerosis	(19)	
Atherosclerosis	CCR2^−/−^, apoE^−/−^ mice	Selective deletion of CCR2 significantly reduced lesion formation of apoE^-/-^ mice	(20)	
Atherosclerosis	Human atherosclerotic plaque samples	CCL2 of human atherosclerotic plaques was significantly related to plaque vulnerability characteristics	(21)	
Stroke	Healthy adults	Genetic susceptibility to increased circulating CCL2 levels was related to a higher risk of stroke	(22)	
Atherosclerosis	ApoE^-/-^ mice with MI	Monocyte-targeted RNAi with CCR2 as a target improved infarct healing in atherosclerosis-prone mice	(23)	
MI	CCL2^-/-^ mice/CCL2 antibody neutralization mice with reperfused MI	CCL2 regulated inflammatory responses essential for MI repair	(24)	
MI	RAW 264.7 cells Mice with MI	Lipid micelles loaded with CCR2 inhibitors affected inflammatory cell migration and cardiac function after MI	(25)	
Ischemic preconditioning	CCL2 TG mice with Coronary artery occlusion and reperfusion	Cardiac overexpression of CCL2 simulated ischemic preconditioning *via* the activation of SAPK/JNK1/2	(26)	
MI	MHC/MCP-1 mice with MI	CCL2 overexpression in the heart prevented cardiac dysfunction and remodeling after MI	(27)	
Hypertension				
Hypertension	CCR2-deficient mice injected with Ang II	CCR2 was required for macrophage infiltration and vascular hypertrophy in ang II-induced hypertension	(10)	
Hypertension	CCR2^-/-^mice/BMT-CCR2^-/-^ mice injected with Ang II	CCR2 played a key role in hypertension-induced vascular inflammation and remodeling	(28)	
Hypertension	RASMCs treated with Ang II	Ang II directly stimulated the expression of the CCL2 gene in the vascular system *via* the AT1 receptor	(29)	
Arterial hypertension	Caucasian patients with primary Arterial hypertension	CCL2 was observed to be increased in hypertensive patients and correlated with the extent of organ injury	(30)	
Salt-sensitive hypertension	TRPV1^-/-^ mice receiving DOCA-salt with vehicle	Enhanced CCL2-CCR2 signaling pathway exacerbated renal injury in patients with salt-sensitive hypertension	(31)	
Renovascular hypertension	CCL2 KO mice with renal artery stenosis	CCL2 is a key mediator of chronic kidney injury in renovascular hypertension	(32)	
Salt-sensitive hypertension	Dahl SS rats with high salt diet	CCL2 mediated early renal leukocyte infiltration of salt-sensitive hypertension	(33)	
Myocardial disease				
EAM/Acute myocarditis	Rats injected with Porcine cardiac myosin Acute myocarditis patients	CCL2 played a significant role in the progression of EAM in rats and in the pathogenesis of acute myocarditis in humans	(34)	
EAM	Rats injected with Porcine cardiac myosin	CCL2 promoted the migration and proliferation of monocytes/macrophages in EAM	(35)	
EAM	Mice injected with mixture of cardiac myosin polypeptide	The expression of CCL2 in EAM was upregulated by IL-17 through Act1/TRAF6/TAK1	(36)	
Viral myocarditis	Mice infected with CVB3	Blockade of CCL2 activity protected against CVB3-induced myocarditis by impairing Th1 polarization	(37)	
EAM	CCR2^-/-^ mice injected with Murine cardiac myosin	The CCL2/CCR2 axis played an important role in the induction of EAM	(38)	
DCM	DCM patients with low to moderate impairment of left ventricular function/Patients with severe left ventricular dysfunction	CCL2 contributed to cardiomyocyte injury in DCM by regulating monocyte infiltration and activation	(39)	
DCM	Mice injected with DOX	CCR2 inhibition reduced mobilization of Ly6C^high^ monocytes in bone marrow and improved cardiac inflammation and left ventricular dysfunction	(40)	
HCM	HCM patients	CCL2 was correlated with left ventricular systolic dysfunction in HCM patients and may be involved in its pathogenesis	(41)	
AC	Dsg2^MT^ mice Dsg2^cKO^ mice	CCL2/CCR2 was involved in the regulation of inflammatory and repair processes during the progression of AC	(42)	

Recently, a large epidemiological study highlighted the causal association of the CCL2-CCR2 pathway with cardiovascular disease in humans (22). Given its critical role in the immune inflammatory response, the CCL2-CCR2 axis is recognized as an important physiological modulator and a viable therapeutic target. Although a great deal of preclinical data (44–46) support the important contribution of the CCL2-CCR2 axis in experimental cardiovascular disease, the existing clinical studies (47, 48) have not yielded satisfactory results. The development and clinical application of drugs on the basis of the CCL2-CCR2 axis for the treatment of cardiovascular diseases continue to be challenging.

In this review, the mechanisms of action of the CCL2-CCR2 axis in the development of cardiovascular disease are described. Also we follow the progress of CCL2-CCR2 axis in relation to preclinical and clinical studies of cardiovascular disease. These results elucidate the essential function of the CCL2-CCR2 axis in cardiovascular evolution and its prospective use as a target for therapy.

## Basic information of CCL2-CCR2 axis

Chemokines are small, highly conserved families of secreted proteins composed of cytokines (49), which are responsible for regulating cell movement in response to chemical stimuli (chemotaxis) (50). Chemokines perform its function by associating with G protein-coupled chemokine receptors (GPCRs) (51) thereby affecting a variety of biological processes and disease conditions, such as cancer (52), cardiovascular diseases (9), liver diseases (53), and intestinal diseases (54). Chemokines are classified as four major families, CXC, CC, XC and CX3C, by the arrangement of their amino-terminal (N-terminal) cysteines (51). CC and CXC are the two major subfamilies of chemokines. Among the CC chemokine subfamilies, CCL2 was the first to be discovered and fully investigated (55, 56).

**Figure 1:**
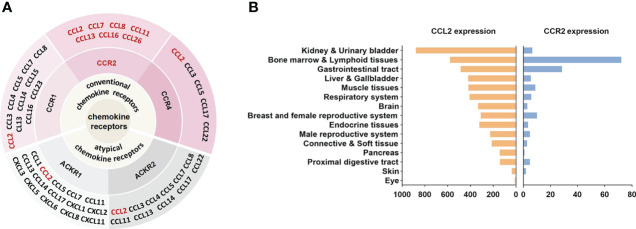
CCL2-CCR2 network and expression in human tissues. **(A)** Chemokines and their receptor networks associated with the CCL2-CCR2 axis (9). The chemokine family is a huge system of ligands and GPCRS. In this network, CCL2 can bind to a variety of receptors, including the classical chemokine receptors (shaded in pink) CCR1, CCR2, CCR4 and the atypical chemokine receptors (shaded in gray) ACKR1, ACKR2. CCR2 can also bind to a variety of ligands (in red), including CCL2, CCL7, CCL8, CCL11, CCL13, CCL16, CCL26. **(B)** CCL2 and CCR2 RNA expression in human tissues, X-axis represents the consensus data based on normalized expression (nTPM) values (data from https://www.proteinatlas.org).

CCL2, also known as monocyte chemoattractant protein-1 (MCP-1), was originally obtained in 1989 from the culture supernatant of human glioma cells and human blood mononuclear leukocytes (57, 58). As a protein consisting of 76 amino acids at 13 kDa, CCL2 has two adjacent amino-terminal cysteine residues (59). CCL2 is primarily secreted by immune cells, in addition, smooth muscle cells, endothelial cells, thylakoid cells, and fibroblasts are also capable of producing CCL2 (58, 60, 61). The expression of CCL2 can be either persistent or inducible. A variety of mediators can induce CCL2 expression, such as IL-1, IL-4, IL-6, TNF-α, TGF-β, IFN-γ, etc (62, 63). CCL2 regulates the migration and infiltration of a wide range of immune cells, including monocytes, macrophages, memory T lymphocytes, and natural killer (NK) cells (50, 59). It has been reported that CCL2 can bind to a variety of receptors **(** **Figure 1A** **)**. For example, by binding to CCR2, CCL2 coordinates inflammatory monocyte transport among bone marrow, circulating and atherosclerotic plaques (9). CCL2 binding to CCR4 activates myosin light chain (MLC) phosphorylation and regulates cell motility and tumor metastasis (64). Furthermore, CCL2 can be bound to atypical chemokine receptors (ACKRs), including ACKR1 and ACKR2, which do not induce cell migration but can alter chemokine gradients (65). Although CCL2 can bind to a variety of receptors, CCR2 is still considered to be the primary receptor for CCL2. The amino-terminal region of CCL2 is an important factor in determining CCR2 affinity for binding and signal selectivity (66).

Since its discovery in 1994 (67), CCR2 has been extensively investigated as a prospective therapeutic target for many diseases. CCR2 belongs to GPCRs, whose steric structure contains the binding sites of G proteins (guanylate-binding proteins) and seven transmembrane α-helices. Depending on the carboxyl terminus, CCR2 is divided into CCR2A and CCR2B (68), which perform different roles. Notably, as with CCL2, CCR2 can also bind to other chemokines, including CCL7, CCL8, CCL11, CCL13, CCL16, and CCL26 (9) **(** **Figure 1A** **)**. These chemokines perform important roles in a variety of diseases. For example, CCL8 was involved in the recruitment of tumour-associated macrophages by hypoxic cervical cancer cells through its binding to CCR2 (69). CCL11 promoted ovarian cancer growth and metastasis by stimulating the proliferation and migration of ovarian carcinoma cell lines (70). CCL13 levels were significantly increased in patients with asthma and allergic rhinitis (71). Interestingly, unlike other chemokines, CCL7 played different or even opposite roles in inflammation. On the one hand, CCL7 was involved in the recruitment of pro-inflammatory mononuclear phagocytes into the local colonic tissue of colitis and could promote acute lung inflammation by recruiting neutrophils (72, 73). On the other hand, cleaved CCL7 was found to act as a general chemokine antagonist that inhibits inflammation (74). In addition, chemokine receptors such as CCR5 and CXCR4 can also interact with CCR2 (51). CCR2 is located in a range of tissues such as heart, liver, spleen, lung, kidney, pancreas, ovary, thymus, brain, blood and spinal cord (75) **(** **Figure 1B** **)**and is broadly expressed in various cells.

**Figure 2:**
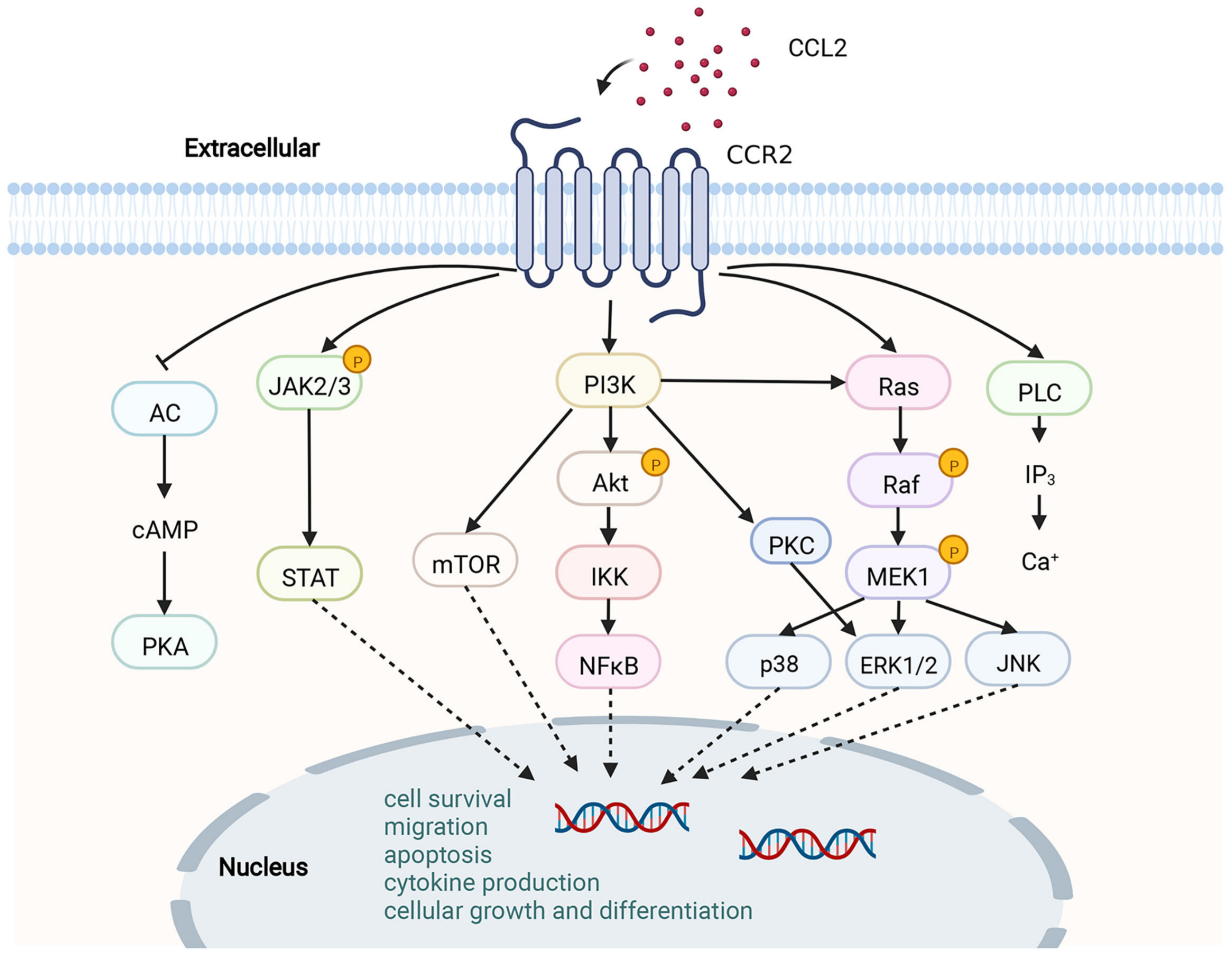
Schematic diagram of the CCL2-CCR2 axis and its signaling pathway. CCR2 is a G PCR. Upon binding of CCR2 to CCL2, a range of signaling downstream is activated, for instance, the JAK/STAT pathway, MAPK pathway and PI3K/Akt pathway. A variety of transcription factors and genes are then activated to participate in cytokine production, cell growth and differentiation, cell survival, migration and apoptosis.This figure was created with BioRender.com.

By binding to CCL2, CCR2 undergoes dimerization and internalization, inducing the expression of monocyte chemotactic protein-1 inducible protein-1 (MCPIP1). Subsequently, transcription and expression of IL-1, CCL2 and TNF genes are initiated by MCP1P1 (76). Upon triggering of CCR2 by CCL2, a variety of intracellular G protein-mediated signaling gateways will be initiated, for instance JAK/STAT, PI3K/MAPKs and PI3K/Akt/ERK/NF-κB (77) **(** **Figure 2** **)**. Activation of these signaling pathways results in the mobilization of multiple transcription factors and genes involved in cytokine production, cell growth and differentiation, cell survival, migration and apoptosis, angiogenesis and inflammation (62, 78, 79). CCL2-CCR2 axis is associated with the advancement of several disorders, like atherosclerosis (9), acute liver failure (80), rheumatoid arthritis (81), pulmonary hypertension (82), diabetes and complications (83), and cancer (78). As a result, CCL2-CCR2 axis is recognized as a potential targeted site for the management of such diseases (1, 2). The CCL2-CCR2 axis has received a lot of attention along with CCL2-CCR2 antagonists (**Table 2**).

**Table 2:** Antagonists of CCL2 and CCR2.

Drug	Target	Conditions	Stage	NCT number/References
Nox-E36	CCL2	Type 2 Diabetes Mellitus (T2DM) Renal Impairment Chronic Inflammatory Diseases Systemic Lupus Erythematosus	I/II	NCT01547897 NCT01372124 NCT00976729
AZD-2423	CCR2	Chronic Obstructive Pulmonary Disease (COPD) Nerve Pain	II	NCT01215279 NCT01200524
BMS-687681	CCR2, CCR5	Pancreatic ductal adenocarcinoma	Pre	(84)
BMS-741672	CCR2	T2DM Neuropathic Pain	II	NCT00699790 NCT00683423
BMS-813160	CCR2, CCR5	Diabetic Kidney Disease Colorectal Cancer, Pancreatic Cancer Lung Cancer, Hepatocellular Carcinoma	I/II	NCT01752985 NCT03184870 NCT04123379
CCX140-B	CCR2	Glomerulosclerosis Diabetic Nephropathy, T2DM	II	NCT03536754 NCT01447147
CCX872-B	CCR2	Pancreatic Cancer	I/II	NCT03778879 NCT02345408
Cenicriviroc	CCR2, CCR5	Primary Sclerosing Cholangitis Prediabetic State, T2DM HIV Non-alcoholic Steatohepatitis (NASH) COVID 19	I/II/III	NCT02653625 NCT02330549 NCT01827540 NCT03517540 NCT04593940
CNTX-6970	CCR2	Chronic Pain, Nociceptive Pain Knee Osteoarthritis	I/II	NCT03787004 NCT05025787
GSK1344386B	CCR2	Atherosclerosis	Pre	(85)
INCB-3284	CCR2	Rheumatoid Arthritis (RA), T2DM	I/II	(86, 87)
INCB-3344	CCR2	Atherosclerosis, Diabetic nephropathy Chronic inflammatory diseases	Pre	(88–90)
INCB-8696	CCR2	Multiple sclerosis (MS), Lupus	I	(86)
INCB-8761	CCR2	Metastatic Pancreatic Ductal Adenocarcinoma Osteoarthritis Chronic Hepatitis C	II	NCT02732938 NCT00689273 NCT01226797
JNJ-17166864	CCR2	Allergic Rhinitis	II	NCT00604123
JNJ-27141491	CCR2	MS	Pre	(91)
JNJ-41443532	CCR2	T2DM	II	NCT01230749
MK-0812	CCR2	MS Arthritis, Rheumatoid	II	NCT00239655 NCT00542022
PF-04634817	CCR2, CCR5	Renal Insufficiency Diabetic Nephropathy Macular Edema, Diabetic	I/II	NCT01791855 NCT01712061 NCT01994291
PQ50	CCR2	Critical Limb Ischemia	II	NCT01232673
RAP-103	CCR2, CCR5, CCR8	Diabetic neuropathic pain	Pre	(92, 93)
RO5234444	CCR2	T2DM	Pre	(94)
RS102895	CCR2, Human ADRA1A, Human ADRA1D, Rat HTR1A	Diabetic nephropathy, Ischemia/reperfusion injury, Hypertension	Pre	(95–97)
RS504393	CCR2, Human ADRA1A, Human ADRA1D, Rat HTR1A	Hypertension, Cardiac Hypertrophy Pain	Pre	(31, 98, 99)
SSR150106	CCR2	RA	II	NCT00545454
TLK-19705	CCR2, CCR5	Atherosclerosis	Pre	(100)

Diabetic nephropathy is one of the most important comorbidities in diabetic patients.

## CCL2-CCR2 axis and cardiovascular disease

### CCL2-CCR2 axis and heart failure

As a complex clinical syndrome, heart failure (HF) is characterized by signs and symptoms caused by any structural or functional disorder of ventricular filling or ejection (101). HF can occur as a result of valvular heart disease, hypertension, ischemic heart disease, and myocardial infarction (MI) (101). A great deal of evidence suggests that the onset and progression of HF is intimately associated with inflammation, no matter the underlying etiology (102, 103). The induction and activation of chemokines is one of the features of the inflammatory response in the failing heart (104). The chemokine CCL2 protein is localized to cardiomyocytes, vascular endothelial and smooth muscle cells, interstitial fibroblasts and infiltrating leukocytes in aortic-cavernous fistula (ACF) rats and is involved in the progression of myocardial dysfunction and HF in ACF rats, with a positive correlation between its expression and the severity of congestive heart failure (CHF) (8). In addition, it is possible that CCL2 myocardial damage, fibroblastic remodeling, and malfunction by stimulating the recruitments of proinflammatory leukocytes in the failing heart (104). In a left ventricular pressure overload model, CCL2-mediated macrophage aggregation acted on myocardial fibrosis *via* a TGF-β-mediated process. Neutralization of CCL2 inhibited macrophage aggregation, TGF-β induction, and fibroblast proliferation, while attenuating diastolic dysfunction and reducing myocardial fibrosis (12). The desmin-deficient mice (des^-/-^) is a progressive HF model characterized by galectin-3 overexpression, a spontaneous inflammatory response that maintains fibrosis, and cardiomyocyte death. This model is accompanied by macrophage infiltration and upregulation of the deleterious macrophage-associated genes CCR2 and Arg1. During the development of des^-/-^ cardiomyopathy, Galectin-3 deficiency downregulated CCR2, Arg1 and pro-fibrotic gene expression as well as amelioration of poor cardiac remodeling, inflammation and failure (13).

In 2000, J.K. Damås et al. first demonstrated the expression and protein localization of chemokines and their receptor genes in the human myocardium (14). Activated platelets significantly inhibit the release of CCL2 from human umbilical vein endothelial cells in a CD154 dependent manner (15). Serum CCL2 levels were measured in 50 CHF patients and showed that serum CCL2 levels were substantially elevated in patients in CHF compared with health controls and were positively related to the seriousness of symptoms as well as the degree of left heart insufficiency (15). CCL2 is significantly correlated with poor prognosis in patients with advanced HF (16).

### CCL2-CCR2 axis with atherosclerosis and coronary artery atherosclerotic heart disease

**Figure 3:**
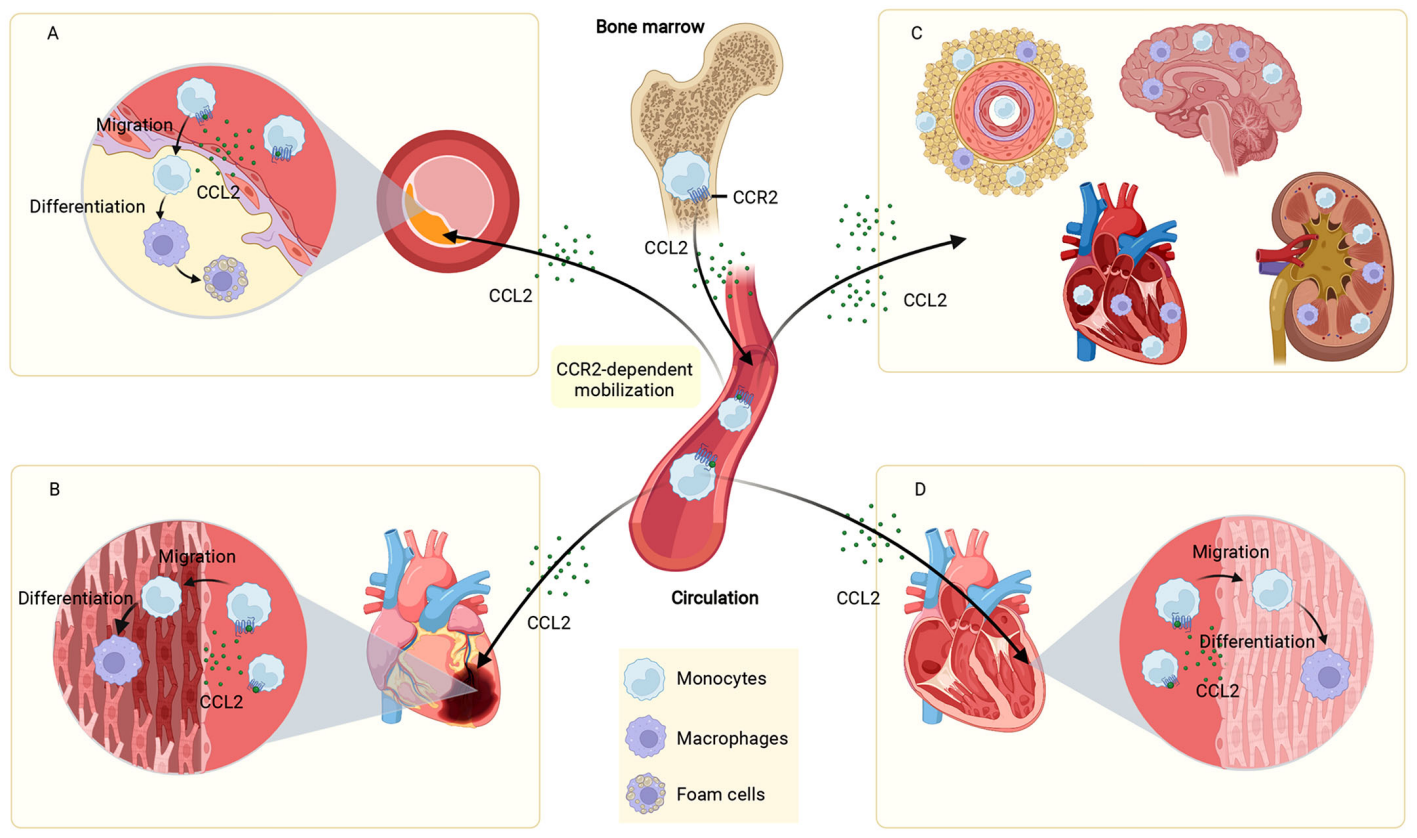
Role of the CCL2-CCR2 axis in cardiovascular disease. The CCL2-CCR2 axis fosters monocyte mobilization from the bone marrow and recruits circulating monocytes to the site of the lesion. In plaques, monocytes are differentiated to macrophages, which proliferate, become foam cells, and coordinate the inflammatory response **(A)**. In infarcted myocardial tissue, monocytes are recruited and differentiated into macrophages that influence MI disease progression **(B)**. In hypertension, monocytes infiltrate into the vascular adventitia, perivascular fat, heart, kidney, and brain, and are involved in elevated blood pressure and end-organ damage **(C)**. In cardiomyopathies (dilated cardiomyopathy as an example), monocytes infiltrate and differentiate into macrophages in the damaged myocardium and participate in the development and progression of cardiomyopathies **(D)**. This figure was created with BioRender.com.

Atherosclerosis underlies the underlying pathophysiology of several cardiovascular diseases. The pathogenesis of atherosclerosis is complicated, and a great deal of data indicates that inflammation has an essential role in the occurrence and progression of atherosclerotic disease (105). CCL2-induced migration of monocytes to the vessel wall is a key activity contributing to the development of atherosclerosis. In this process, NF-κB was involved in regulating the expression of CCL2 (17). As early as 1991, it was found that CCL2 expression was more readily detected in different regions of atherosclerotic plaques compared to normal vessels (18). TNF-a, IFN-γ and mixed lymphocyte culture supernatants were all demonstrated to stimulate the production of CCL2 (18). In contrast, lack of CCL2 decreased the atherosclerosis of low-density lipoprotein receptor-deficient mice (19). CCR2 was identified as a genetic determinant of atherosclerosis in mice (20). Selective deletion of CCR2 significantly decreased atherosclerotic lesion formation and reduced macrophage accumulation in plaques in ApoE knockout (Apoe^-/-^) mice (20). Lipid-laden foam cells are a hallmark of atherosclerosis (106). Under the action of the CCL2-CCR2 axis, circulating monocytes are recruited to atherosclerotic plaques and differentiate into macrophages, which proliferate, become foam cells, and orchestrate the inflammatory response (107). All these evidences suggest that CCL2-CCR2 is closely associated with atherosclerotic heart disease **(** **Figure 3A** **)**.

Recently, a team studied atherosclerotic plaques in 1199 patients treated with endarterectomy for carotid stenosis. It was found that CCL2 of human atherosclerotic plaques was significantly related to plaque vulnerability characteristics, as evidenced by the fact that CCL2 levels were correlated with plaque matrix turnover, pro-inflammatory plaque characteristics, plaque vulnerability pathological histological features, clinical plaque instability, and perioperative vascular events 30 days after plaque removal. This large-scale human study expands earlier epidemiological, genetic, as well as experimental research (21). However, consistent with our recognition that cross-sectional studies do not allow for causal inference, this study cannot explain the cause-and-effect role of CCL2 in human atherosclerosis. Significantly, another two-sample Mendelian randomization study explored this causal relationship, showing that higher circulating CCL2 levels were related to a greater degree of risk for ischemic stroke, especially large artery stroke, as well as coronary artery disease and MI (22).

Ischaemic heart disease is the major reason for death worldwide (1). Although the rational use of drugs and advances in reperfusion therapy have significantly reduced acute mortality after MI in patients, they have put patients who survive at greater risk of developing chronic heart failure (CHF) (108). The adult mammalian heart is virtually incapable of regeneration and inflammation-driven scar formation contributes to the repair of the infarcted heart (109). Excessive or insufficient acute inflammation after MI plays a key role in the process of ventricular remodeling (110, 111). Studies have shown that chemokines regulate the serial mobilization of immune cell subsets in the infarcted heart, with CC chemokines (e.g., CCL2) mediating monocyte recruitment (109, 112) **(** **Figure 3B** **)**. Knockdown of CCR2 reduces the recruitment of Ly-6C^high^ monocytes to the infarct site, alleviates infarct inflammation, and inhibits post-infarction myocardial left ventricular remodeling, thereby promoting myocardial infarct healing (23). In addition to the widely recognized role of monocyte chemotaxis, CCL2 is also involved in regulating the phenotype and activity of monocytes. CCL2^-/-^ mice have significantly reduced levels of goat antibone bridge protein (OPN)-1 expression in infarct-zone macrophages, decreased activation of macrophages and infiltration of myofibroblasts. These mechanisms, while attenuating left ventricular remodeling after MI, are accompanied by a prolonged inflammatory phase, a impaired phagocytosis of damaged cardiomyocytes and late replacements of injured cardiomyocytes by sarcomeres, among other undesirable consequences (24). Interestingly, although the results of many MI animal experiments showed that disruption of the CCL2/CCR2 axis reduced myocardial infarct size (25, 113), not all studies had the same conclusion. The percentage of infarct area in transgenic mice with cardiomyocyte-specific overexpression of CCL2 was lower than in normal controls after ischemia/reperfusion. Cardiomyocyte overexpression of CCL2 resulted in chronic infiltration and activation of leukocytes, which led to increased secretion of TNF-a and activation of SAPK/JNK1/2, ultimately achieving cardioprotective effects through activation of MAPKs (26). In addition, myocardial CCL2 overexpression prevented left ventricular dysfunction and remodeling after MI by inducing infiltration of macrophages, secretion of myocardial IL-6, accumulation of myocardial fibroblasts, and neovascularization (27).

### CCL2-CCR2 axis and hypertension

Hypertensive disorders are responsible for a variety of serious complications including hypertensive heart disease, stroke and renal failure. The inflammatory response in the arterial wall contributes to the occurrence and maintaining of hypertension (114). CCL2 is considered to be an essential mediator of the inflammatory response in the arterial vascular system.The CCL2/CCR2 axis has been shown to play a critical role in vascular inflammation, vascular remodeling, and vascular hypertrophy *via* monocyte infiltration and macrophage recruitment in a mouse model of hypertension (10, 28). Angiotensin II (Ang II) directly stimulated the expression of the CCL2 gene in the vascular system *via* the Ang II type-1 (AT_1_) receptor (29). CCL2 expression was significantly elevated in the aortic tissue of animals suffering from hypertension after Ang II injection (115). CCR2 played a crucial role in macrophage infiltration, vascular hypertrophy, inflammation and remodeling in animal models of Ang II-induced hypertension. Hypertension-induced infiltration of arterial wall macrophages was almost abolished and vascular hypertrophy was significantly decreased in CCR2-deficient mice (10). Ang II-induced inflammation and remodeling of blood vessels was significantly attenuated in CCR2^-/-^ mice and bone marrow-transferred mice with a leukocyte-selective CCR2 deficiency (BMT-CCR2^-/-^) (28). In clinical practice, serum CCL2 was measured in 740 hypertensive patients, and soluble CCL2 was observed to be increased in hypertensive patients and correlated with the extent of organ injury (30). In addition, the expression of CCR2 was elevated on the surface of monocytes in hypertensive patients and decreased after treatment by Ang II receptor blockers (28).

CCL2-CCR2 plays an instrumental part in the progression of experimental hypertensive kidney damage. Enhanced CCL2-CCR2 signaling pathway exacerbated renal injury in patients with salt-sensitive hypertension (31). Lack of CCL2 ameliorated renal cortical atrophy and reduced the number of infiltrating monocytes as well as CCR2, CD206, CCL5, CCL7 and CCL8 expression, thereby preventing chronic kidney injury in mice with renal vascular hypertension (32). Blockade of CCR2 using the CCR2 antagonist RS102895 prevented renal leukocyte infiltration early after a high salt diet and attenuated salt-sensitive hypertension and renal injury, thus CCL2-CCR2 is considered a prospective pathway to alter renal leukocyte infiltration and lead to salt-sensitive hypertension (33).

### CCL2-CCR2 axis and myocardial disease

Features by inflammatory cell infiltration of the heart and subsequent deterioration of its function, myocarditis is an important factor in chronic dilated cardiomyopathy (DCM), acute HF and sudden death (116). The expression of CCL2 was raised in the heart and serum of rats with experimental autoimmune myocarditis (EAM) from the acute to the recovery phase, which was highly correlated with the expression levels of TNF-a, IL-1β and IL-6 (34, 35). Regulation the inflammatory infiltration of EAM by adjusting CCL2 expression through Act1/TRAF6/TAK1 is one of the pathological mechanisms of myocarditis (36). *In vivo* blockade of CCL2 activity reduced the severity of CVB3-induced myocarditis, and the main mechanism may be related to effective inhibition of chemotaxis and reduction of systemic and local Th1 immune responses (37). EAM mice lacking CCR2 have decreased myocarditis severity (38). siRNA silencing of CCR2 (SiCCR2) decreased the number of Ly6C^high^ monocytes and migration of bone marrow granulocyte macrophage precursor cells to the blood in the hearts of mice with acute autoimmune myocarditis, prevented macrophage magnetic resonance imaging enhancement, and improved ejection fraction (11). In clinical studies, elevated CCL2 levels at first occurrence in patients with acute myocarditis were dramatically related to severity of disease and prognosis. This was demonstrated by significantly higher serum CCL2 levels in patients with acute myocarditis on admission than in healthy volunteers and significantly higher serum CCL2 levels in patients who died of acute myocarditis than in surviving patients (34). Cardiac tissue biopsy samples from patients with myocarditis were enriched for CCR2^+^ cells and had elevated CCL2 and CCR2 mRNA expression compared to control specimens obtained from individuals dying from trauma with no history of cardiac disease (11).

DCM is the most common non-ischemic cardiomyopathy leading to HF. CCL2 expression is upregulated in late stages of DCM and negatively correlates with left heart function, a mechanism that may lead to cardiomyocyte injury through infiltration and activation of monocytes (39). Targeting CCR2 to downregulate its protein expression inhibited the mobilization of Ly6C^high^ monocytes in the bone marrow, thereby improving cardiac systolic functions as well as reducing ventricular remodeling in mice with DCM caused by low doses of adriamycin (DOX) (40). Levels of CCL2 were also dramatically increased in the serum and myocardium of patients with hypertrophic cardiomyopathy (HCM) and were related to left ventricular systolic dysfunction (41).

Arrhythmogenic cardiomyopathy (AC) is a hereditary disease described by arrhythmias, fibrosis and cardiac dilatation. AC is a primary disease of the myocardium that can cause cardiac sudden death as well as HF (117). The pathogenesis of AC is currently not well defined. It was revealed that particular immune cell groups along with chemokine expression profiles regulate inflammatory and reparative processes during the whole course of AC progression. Among them, CCL2 and CCR2 mRNA are upregulated during disease onset, acute and chronic phases, and the early phase of AC is accompanied by a reaggregation of CCR2^+^ inflammatory monocytes to the heart (42).

## Drug studies based on the CCL2-CCR2 axis for the treatment of cardiovascular diseases

### Preclinical studies

#### Drugs targeting CCL2

11K2 is an inhibitory monoclonal antibody with high affinity for human CCL2 and a convenient cross-reactivity with mouse CCL2 and CCL12. In the presence of 11K2, ApoE^-/-^ mice show a reduction in plaque area, a decrement in macrophage and CD45^+^ cell numbers, and an improvement in collagen content, resulting in a consistent plaque phenotype (118).

Bindarit (BIN), a small molecule with anti-inflammatory activity in a variety of inflammatory diseases, selectively inhibited the production of CCL2, CCL7, and CCL8 which played an important role in the homeostatic localization and transport of immune cells (119). The inhibition of chemokines by BIN was probably achieved by interfering with the classical NF-κB pathway (120). Furthermore, in a mouse model of lipopolysaccharide-induced cytokine production, BIN decreased the activity levels of both CCL2 and TNF-α (121). BIN has been shown to be efficacious in inflammatory diseases such as diabetes-associated periodontitis (122), neuritis (123), osteoarthritis (124), autoimmune encephalomyelitis (125) and acute proliferative lupus nephritis (126). Selective inhibition of CCL2 by BIN reduced the chemotactic process of inflammation that persisted at the site of lesions and infections (119, 120). BIN reduced in-stent stenosis in pigs by suppressing the generation of CCL2 (44). The mechanism of action of BIN in controlling *de novo* intima formation and restenosis may be associated with inhibition of CCL2 and CCL7 generation and induction of smooth muscle cell differentiation in human coronary arteries (127). However, the non-specific distribution *in vivo* limits the application of BIN in atherosclerosis. A yeast-derived microcapsule-mediated nano-drug delivery approach delivers BIN to the interior of atherosclerotic mouse plaques, significantly enhancing the inhibitory effect of CCL2 and further reducing the recruitment of monocytes to atherosclerotic plaques (128).

In addition, gene therapy strategies have opened a new window for CCL2-CCR2 treatment of cardiovascular disease. The CCL2 mutant 7ND with a 7 amino acid deletion at the N-terminal end functions as a dominant-negative inhibitor of CCL2. Monocyte activation and infiltration following arterial injury and experimental restenosis following balloon injury and stent placement is inhibited by 7ND gene transfer. In addition, 7ND gene transfer improved platelet stability and limited the development of early atherosclerotic lesions in hypercholesterolemic mice as well as the progression of pre-existing atherosclerotic lesions (129, 130). In a similar vein, Liehn E (45) et al. showed that the non-excited CCL2 mutant PA508 inhibits monocyte chemotaxis or transendothelial migration to CCL2 by competing with CCL2 to interfere with its presentation. Although PA508 had no effect on leukocyte sorting, levels of CCL2, nor organ function or morphology in wild-type mice, it resulted in reduced recruitment of inflammatory leukocytes, demonstrating specific inhibition of the CCL2-CCR2 axis. In addition, PA508 showed good effects in two of the most common mouse models of cardiovascular disease. In a hyperlipidemic ApoE^-/-^ mouse model, PA508 significantly reduced intimal plaque area and infiltration of individual nucleated cells in mouse carotid arteries and increased the content of vascular endothelial cells. In a myocardial ischemia/reperfusion mouse model, PA508 substantially decreased myocardial infarct area, monocyte infiltration, collagen and myofibroblast levels in the infarcted region, and protected cardiac function in mice.

#### Drugs targeting CCR2

RS102895 (IC_50_ = 360nM) and RS504393 (IC_50_= 89nM) are potent CCR2b inhibitors. In addition to CCR2, it also inhibits human ADRA1A (IC_50_= 130nM/72nM), ADRA1D (IC_50_= 320nM/460nM), and rat HTR1A (IC_50_= 470nM/1070nM) in cells (131). RS102895 inhibited ischemia/reperfusion-induced cardiomyocyte apoptosis, and pretreatment with RS102895 restored reduced cell viability after ischemia/reperfusion (95). In addition, RS102895 blocks the infiltration of renal leukocytes in the early stages of salt-sensitive hypertension (33) and exerts anti-inflammatory and renoprotective effects in hypertension (96, 132). Notably, an interesting study (133) demonstrated the important role of circadian rhythmic leukocyte recruitment in atherosclerosis and established the CCL2-CCR2 axis as its modulator. A chronopharmacological treatment strategy based on RS102895 was effective in inhibiting the development of early atherosclerotic lesions while not affecting the inflammatory process of microcirculation. In response to RS504393, DOCA-salt hypertensive rats showed reduced monocyte/macrophage infiltration and chemokine/cytokine production, decreased NF-κB activity, and improved renal dysfunction as well as morphological impairment (31). Early blockade of CCR2 with RS504393 after transverse aortic constriction (TAC) reduced CCR2^+^ cardiac macrophage levels, inhibited VCAM expression, and improved late left ventricular dysfunction and cardiac fibrosis (98).

Propagermanium (PG) is a CCR2 antagonist with high safety and availability and is often evaluated for its therapeutic potential as a CCR2 antagonist. Studies have shown that PG reduces atherosclerosis in ApoE^-/-^ mice by suppressing macrophage infiltration (46). PG also inhibited the formation of macrophage-mediated coronary atherosclerotic pathology in pigs (134) and atherosclerotic lesions in rabbits with Watanabe heritable hyperlipidemia development (135). Similar to PG, TLK-19705 (IC_50_= 700 nM)also blocks the CCL2/CCR2 signaling pathway. Continuous administration of TLK-19705 over 8 weeks dramatically decreased the area of atherosclerotic lesions in ApoE^-/-^ mice (100). Nevertheless, not all CCR2 inhibitors are therapeutically effective. INCB-3344 is a potent CCR2 antagonist with IC_50_ values of 9.5 nM (mCCR2), 5.1 nM (hCCR2) for antagonistic binding activity and 7.8 nM (mCCR2), 3.8 nM (hCCR2) for antagonistic chemotactic activity (136). Blockade of CCR2 with INCB-3344 did not affect atherosclerotic lesions although it caused a dramatic reduction of Ly-6C^hi^ monocyte subpopulations in the blood of ApoE^-/-^ mice (88). Similarly, GSK1344386B failed to affect total atherosclerotic lesion size although it blocked accelerated monocyte recruitment and macrophage infiltration in an atherosclerotic mouse model (85). These differences may be due to the recognition of receptors other than CCR2. It was reported that PG selectively inhibited not only CCL2-induced chemotaxis but also CCL7-induced monocyte migration by targeting GPI-anchored proteins that are closely related to CCR2 (137). TLK-19705 inhibited CCL4-induced chemotaxis of human peripheral blood mononuclear cells, a chemokine that signals through CCR5 (138).

#### Other drug studies affecting the CCL2-CCR2 axis

Several drugs with broad therapeutic targets have been found to act on the CCL2/CCR2 axis to treat cardiovascular disease. There is growing evidence that the sodium-glucose co-transporter protein 2 (SGLT2) inhibitor, empagliflozin, contributes to the treatment of cardiovascular disease, while reducing HF and cardiovascular mortality among inpatients (139–142). Recent findings showed that engramine significantly downregulated the expression of cardiac macrophage markers Itgax and CCL2, increased the expression of MRc1, decreased the infiltration rate of cardiac macrophages in mice, and improved myocardial structural and cardiac function abnormalities induced by chronic hypercortisolism in mice (143). Many anesthetics have been reported to show cardioprotective effects in clinical practice (144–146). Diazepam is a local anesthetic that is rapidly distributed in the liver, kidneys, lungs, brain, heart and small intestine (147) and has shown cardioprotective potential in a series of studies (148, 149). Diazepam pretreatment effectively decreases the release of proinflammatory cytokines ILs and TNF-α, the expression of CCR2 chemokines, and inhibits oxidative nitrosative stress and apoptosis in myocardial ischemia-reperfused rats, which exerts cardioprotective effects. MicroRNAs (miRs) are small non-coding RNAs that participate in post-transcriptional gene modulation and are critical for cell differentiation, homeostasis and animal development (150, 151). Different miRs have been reported to regulate the formation and progression of atherosclerotic plaques by regulating phagocytosis, and are considered as potential targets of molecules for anti-atherosclerotic therapy (152). Among them, miR-146a and miR-181b effectively inhibited the CCL2, CCL5, CCL8 and CXCL9 as well as monocyte adhesion to endothelial cells by targeting E-selectin, which reduced the size of atherosclerotic plaques and improved endothelial inflammation and atherosclerosis (153).

In addition, some herbal compounds can also exert therapeutic effects on cardiovascular diseases by affecting the CCL2-CCR2 axis. Fufang Zhenzhu Tiao Zhi (FTZ) formula is a traditional herbal remedy for the treatment of disorders of glucose and lipid metabolism (154). FTZ can reduce serum IL-12 and CCL2 levels and cardiac IL-12, IL-6 and CCR2 mRNA levels in streptozotocin-induced diabetes and improve streptozotocin induced diabetic cardiomyopathy (155). Gui Zhi Tang can reduce the levels of IL-6, CCL2, IL-1β, MMP2, and MMP9 in Dahl salt-sensitive rats, reducing the area of myocardial fibrosis and exerting antihypertensive effects (156). Network-based pharmacology to explore the molecular targets of the action of Wenxin Keli (WXKL) in the treatment of atrial fibrillation, it was found that these targets were closely related to inflammatory response, oxidative stress response, and immune regulation, and CCL2 was one of the main targets of its action (157). Bioinformatics analysis suggests that hemiphilin injection may exert a potential protective effect against neocoronary pneumonia, especially COVID19-induced cardiac insufficiency, by targeting seven Hub genes, including CCL2 and CXCL8, to inhibit oxidative stress, prevent atherosclerotic plaque formation, and suppress inflammation and apoptosis (158).

### Clinical studies

#### Clinical trials targeting the CCL2-CCR2 axis

Although pharmacological studies targeting the chemokine signaling pathway have been extensive, there are currently only three marketed drugs based on the chemokine signaling pathway (9, 159). The extensive actions of chemokines in damage and repair make chemokine-based clinical translation challenging. Despite the remarkable efficacy of BIN in cellular and animal models for coronary atherosclerotic heart disease mentioned above, few clinical studies have been conducted around BIN. Results from a phase II trial showed that BIN was well tolerated and may have a protective effect on the vessel wall after angioplasty, but this study did not meet the primary endpoint and was considered a negative study (48). The clinical efficacy of BIN in the treatment of cardiovascular disease remains to be further validated.

MLN1202 is a monoclonal antibody designed to interact with CCR2 and inhibits CCL2 binding in a highly specific manner. A phase II trial of MLN1202 showed a substantial decrease in circulating levels of highly sensitive C-reactive protein in patients with atherosclerotic cardiovascular disease after 4 weeks of treatment with MLN1202, which lasted for 8 weeks. However, the study was limited in size and insufficient to stratify baseline covariates that might confound the results (47). In addition, no phase III trials of this project have been reported.

#### Other relevant clinical studies affecting the CCL2-CCR2 axis

Colchicine is a novel and complex anti-inflammatory agent which has been documented in medical literature as early as approximately 1550 B.C (160). In the past few years, several clinical trials have evidenced the function of colchicine in cardiovascular disease (161–163). In 2019, Tucker et al. (164) reported firstly the effect of colchicine on transcoronary (TC) chemokine levels in acute coronary syndrome (ACS) patients. In this open-label clinical trial, 13 patients with stable angina pectoris (SAP) and 12 patients with ACS were treated with colchicine, while 13 additional patients with ACS did not receive treatment. According to the results, CCL2 and CX3CL1 levels were elevated in patients with ACS compared with patients with stable coronary artery disease, and TC levels of serum CCL2, CCL5, and CX3CL1 were significantly reduced by colchicine treatment in patients with ACS. In addition, colchicine inhibited the expression of CCL2 gene in monocytes isolated from healthy donors. The above results suggest that colchicine inhibits the expression of chemokines such as CCL2 in patients with ACS, thereby suppressing the migration of monocytes. However, only 38 patients were included in this study, and further large-scale clinical trials are required to demonstrate the mechanism of action of colchicine based on the CCL2 pathway in the treatment of cardiovascular disease.

## Discussion

Cardiovascular disease is closely related to inflammation, and recently, significant progress has been made in studies related to inflammation-based treatment of cardiovascular disease. Unlike canakinumab (5) and colchicine (6), which were effective in reducing the inflammatory response to cardiovascular disease, in a Cardiovascular Inflammation Reduction Trial involving 4786 patients at high risk for cardiovascular events, low-dose methotrexate did not reduce cardiovascular risk in patients (165). The results of these trials emphasize the importance of selecting the appropriate anti-inflammatory pathway and drug candidates in the treatment of cardiovascular disease.

Studies on animals have largely suggested that the CCL2-CCR2 axis is involved in disease processes.CCL2 and CCR2 knockout mice provide convincing evidence for a role of the CCL2-CCR2 axis in monocyte chemotaxis and inflammation (166, 167). A wealth of genetic, epidemiological and experimental data supports the causality of the CCL2-CCR2 axis in cardiovascular disease. Although pharmacologic targets for the CCL2-CCR2 axis in pre-clinical model of cardiovascular disease have been highly effective, clinical outcomes based on the CCL2-CCR2 axis for the treatment of cardiovascular disease have been disappointing to date (**Table 3**). This may be associated with the complexity of the CCL2 and CCR2 molecular structures, the difficulty in choosing the best target between CCL2 and CCR2, the confounding of the CCL2-CCR2 axis, the physiological circadian variation, and the somatic side impacts of CCL2-CCR2-targeted macromolecules (9). It is clear that these issues must be considered to achieve further breakthroughs in clinical applications. Therefore, more in-depth mechanistic studies and clinical studies in larger cohorts are needed before we can successfully design CCL2-CCR2-targeted therapies to significantly alleviate cardiovascular disease.

**Table 3:** Drug studies targeting the CCL2-CCR2 axis for the treatment of cardiovascular disease.

Drug	Type of drug	Target	Nature of action	Conditions	Stage	NCT number/References
11K2	Monoclonal antibody	CCL2, CCL12	CCL2/CCL12 inhibitory antibody	Atherosclerosis	Pre	(118)
Bindarit	Small molecule	NF-κB	CCL2/CCL7/CCL8 inhibitor	Atherosclerosis, Coronary stent restenosis	Pre II	(44, 127, 128) NCT01269242
7ND	Recombinant CCL2 variant	CCL2	CCL2 Dominant-negative inhibitor	Atherosclerosis, Experimental restenosis, Vascular remodeling after injury	Pre	(129, 130)
PA508	Recombinant CCL2 variant	CCL2	CCL2 competitor	Atherosclerosis, Myocardial ischemia/ reperfusion injury	Pre	(45)
RS102895	Small molecule	CCR2, Human ADRA1A, Human ADRA1D, Rat HTR1A	CCR2/Human ADRA1A/Human ADRA1D/Rat HTR1A antagonist	Ischemia/reperfusion injury, Hypertension	Pre	(33, 95, 96, 132)
RS504393	Small molecule	CCR2, Human ADRA1A, Human ADRA1D, Rat HTR1A	CCR2/Human ADRA1A/Human ADRA1D/Rat HTR1A antagonist	Hypertension, Cardiac Hypertrophy	Pre	(31, 98)
Propagermanium	Organometallics	GPI‐anchored proteins associated to CCR2	CCR2 inhibitor	Atherosclerosis	Pre	(46, 134, 135)
TLK-19705	Small molecule	CCR2, CCR5	CCR2/CCR5 antagonist	Atherosclerosis	Pre	(100)
INCB-3344	Small molecule	CCR2	CCR2 antagonist	Atherosclerosis	Pre	(88)
GSK1344386B	Small molecule	CCR2	CCR2 antagonist	Atherosclerosis	Pre	(85)
MLN1202	Monoclonal antibody	CCR2	CCR2 inhibitory antibody	Atherosclerosis	II	NCT00715169

In conclusion, a wealth of animal and human research has provided evidence that the CCL2-CCR2 axis is important in the progression of cardiovascular disease, and the success of pharmacological targeting studies of the CCL2-CCR2 axis holds promise for a gradual transition to clinical trials. Although the CCL2-CCR2 axis-based treatment of cardiovascular disease may not immediately impact cardiovascular disease therapeutic practice, it does open the door for clinical translation of chemokines and their receptor modulators. More in-depth mechanistic studies and larger cohorts of clinical research directed at the CCL2-CCR2 axis will suggest new approaches for improving the prevention and treatment of cardiovascular disease.

## Author contributions

HZ, KY, FC, QL, WC, YH, and ZL participated in the conception of the study, collection of the paper, and compilation of the data. HZ wrote the original manuscript and prepared the figures. GF, LL, JN, and FH provided constructive comments and revised and proofread the manuscript. All authors contributed to the article and approved the submitted version.

## Funding

This research was funded by Innovation Team and Talents Cultivation Program of National Administration of Traditional Chinese Medicine (No: ZYYCXTD-D-202207), Tianjin Municipal Education Commission Scientific Research Program (2021KJ131).

## Conflict of interest

The authors declare that the research was conducted in the absence of any commercial or financial relationships that could be construed as a potential conflict of interest.

## Publisher’s note

All claims expressed in this article are solely those of the authors and do not necessarily represent those of their affiliated organizations, or those of the publisher, the editors and the reviewers. Any product that may be evaluated in this article, or claim that may be made by its manufacturer, is not guaranteed or endorsed by the publisher.
